# Comparison of embolic agents for varices during transjugular intrahepatic portosystemic shunt for variceal bleeding: Tissue gel or coil?

**DOI:** 10.1016/j.jimed.2020.08.008

**Published:** 2020-08-17

**Authors:** Linfeng Zhou, Binyan Zhong, Hang Du, Wansheng Wang, Jian Shen, Shuai Zhang, Wanci Li, Haohuan Tang, Peng Zhang, Weihao Yang, Xiaoli Zhu

**Affiliations:** a Department of Interventional Radiology, The First Affiliated Hospital of Soochow University, Suzhou, China; b Department of Interventional Radiology, Hubei Cancer Hospital, Hubei Cancer Research Institute, Affiliated Cancer Hospital of Tongji Medical College, Huazhong University of Science and Technology, HuBei, China

**Keywords:** Transjugular intrahepatic portasystemic shunt, Esophageal and gastric varices, Embolotherapy

## Abstract

**Purpose:**

We aimed to compare treatment efficacy, safety and material cost between tissue gel and coil regarding variceal embolization during transjugular intrahepatic portosystemic shunt (TIPS).

Materials & Methods: This retrospective study including cirrhotic patients with variceal bleeding treated with TIPS combined with variceal embolization between January 2016 and August 2017. Patients were divided into three groups according to embolic agents used in variceal embolization: tissue gel group (Group A), combination group (Group B), and coil group (Group C). The primary endpoint was 1-year rebleeding rate after TIPS creation. The secondary endpoints included shunt dysfunction, overt hepatic encephalopathy, liver function, and embolic agents-related expense.

**Results:**

A total of 60 patients (30, 10, and 20 in Group A, B, and C) were included. Variceal rebleeding occurred in 3 (10%), 0 (0%), and 4 (20%) patients within one year after TIPS creation in Group A, B, and C, respectively. Stent dysfunction occurred in 2 (3.3%) patients and 9 (15.0%) patients experienced overt hepatic encephalopathy. No significant differences were observed between three groups regarding primary and secondary endpoints except embolic agents-related expense, with a significantly lower cost in Group A when compared to the other two groups. Stent dysfunction occurred in two patients, with one patient in Group A developed acute occlusion caused by thrombus and another patient in Group C underwent stent stenosis during follow-up.

**Conclusions:**

Compares to coil alone or combines with coil, tissue gel has similar treatment efficacy and safety, but with significantly lower cost for variceal bleeding during TIPS.

## Introduction

With an annual occurrence of approximately 10%–15%, variceal bleeding (VB) is a life-threatening complication in patients with cirrhosis and portal hypertension.^1^ The 6-week mortality is around 15%–20%.^2^^,^^3^ In addition, recurrent VB has a high occurrence rate (1-year rate: > 50%).^2^^,^^3^ The recommended first-line treatment for VB is endoscopic band ligation combined with vasoactive drugs and nonselective beta-blockers. Transjugular intrahepatic portosystemic shunt (TIPS), an interventional radiological technique, is recommended as second-line choice for those with failed first-line treatment.^1^^,^^4^

By connecting the hypertensive portal vein to a normotensive hepatic vein and bypassing the site of increased resistance, TIPS is an effective and well-established technique for treating VB, especially after the introduction of the expanded polytetrafluoroethylene (ePTFE)-covered stent to clinical application.^5^, ^6^, ^7^, ^8^ Theoretically, variceal embolization (VE) during the TIPS procedure is expected to enhance the treatment efficacy because fragile varices are considered a risk factor for hemostatic efficacy and recurrent bleeding.^9^ Several studies have identified that TIPS combined with VE results in better prognosis compared with TIPS alone. Therefore, VE during TIPS is preferred especially for gastric varices.^9^, ^10^, ^11^, ^12^, ^13^, ^14^

Currently, several embolic agents are applied in VE, including tissue gel (N-butyl 2-cyanoacrylate, NBCA), coil, gelatin sponge, and vascular plug.^10^^,^^13^^,^^15^, ^16^, ^17^, ^18^ Regardless of the embolic technique, the overall success rates are high. Among the embolic agents, tissue gel and coil are widely used for VE during TIPS.^10^^,^^13^^,^^15^ Nevertheless, few studies focusing on the treatment efficacy and cost comparison between these two embolic agents have been conducted and reported. Therefore, we performed this retrospective study with the aim of comparing the treatment efficacy and cost of tissue gel and coil for VE during TIPS for VB.

## Materials and methods

### Patient criteria

This retrospective study was approved by the institutional review board at The First Affiliated Hospital of Soochow University. The need for informed consent was waived owing to the retrospective design. The study was performed in accordance with the Declaration of Helsinki. The authors declared that they have no conflicts of interest to this work. We declare that we do not have any commercial or associative interest that represents a conflict of interest in connection with the work submitted. Patients with VB and cirrhotic portal hypertension treated with TIPS combined with VE at The First Affiliated Hospital of Soochow University between January 2016 and August 2017 were screened.

The inclusion criteria for the study were as follows: 1) age 18–85 years with liver cirrhosis diagnosed on the basis of clinical presentations, laboratory tests, imaging studies, or liver biopsies; 2) hemorrhage from varices presenting with gastroesophageal varix (GOV) type 2 and isolated gastric varix (IGV) type 1 confirmed by endoscopy; 3) failure of endoscopic and medical treatment; 4) Child-Turcotte-Pugh (CTP) score ​< ​14 and Model for End-stage Liver Disease score ​< ​18; and 5) successful TIPS creation. The exclusion criteria were as follows: 1) absolute contraindications to TIPS, such as severe cardiopulmonary diseases, renal impairment, severe encephalopathy, and progressive liver failure; 2) uncontrolled systemic infection or sepsis; and 3) liver cancer or other extrahepatic malignancy.

### TIPS procedure

**Fig. 1:**
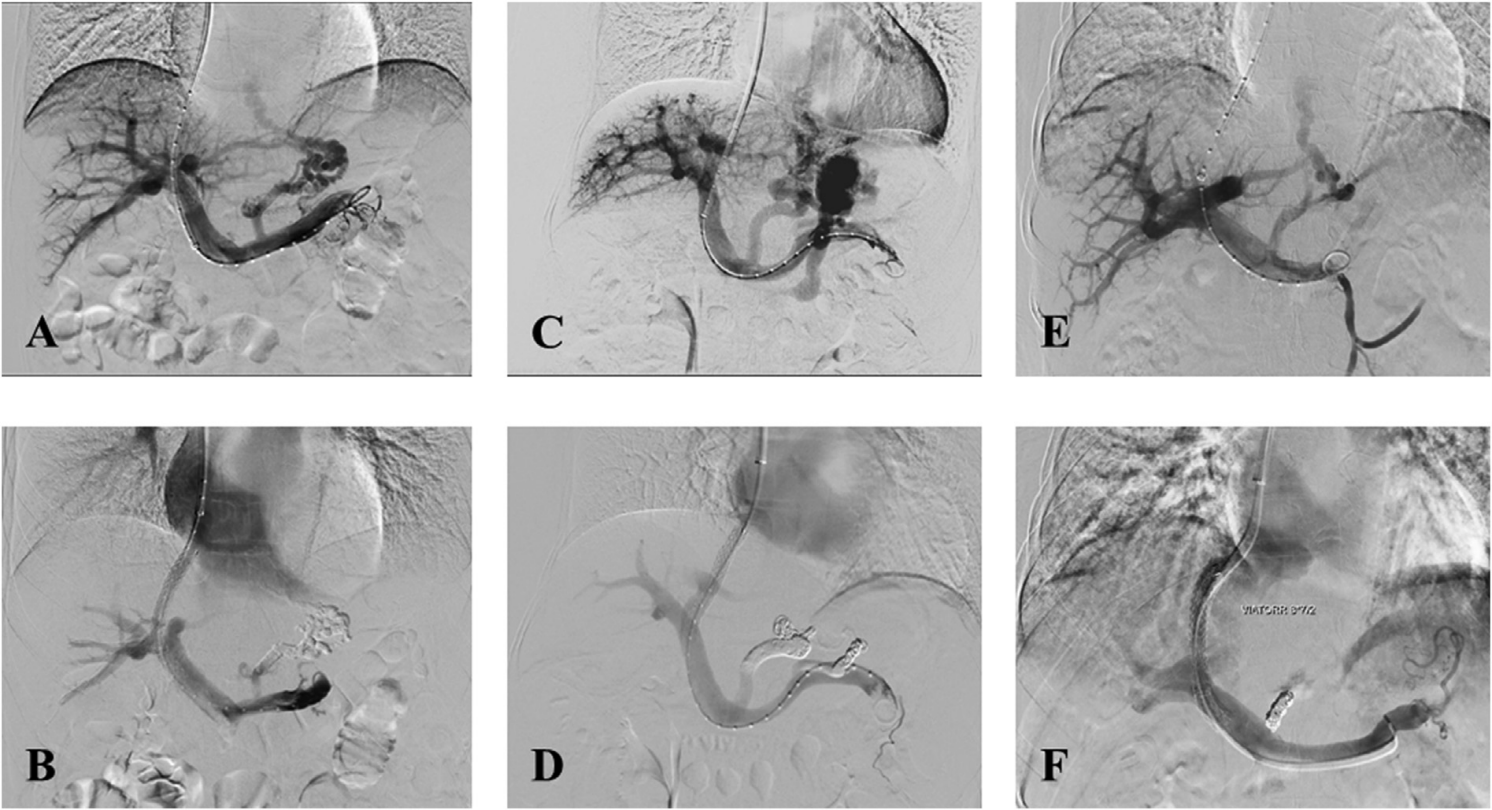
Procedure of TIPS creation. Pre-procedure (A, C, and E) and post-procedure (B, D, and F) venography in patients who underwent embolization with tissue gel (A, B), tissue gel combined with coil (C, D), and coil (E, F).

All TIPS procedures were performed under local anesthesia via a transjugular approach by two interventional radiologists (XLZ with >20 years of experience and another radiologist with >10 years of experience) (Fig. 1). A standard TIPS set (RUPS-100; Cook Medical, Bloomington, IN, USA) was used for TIPS creation. After a successful puncture, portal venography was performed to confirm and assess the category, distribution, and degree of varices. VE was performed before stent insertion, and embolic agents were used according to portal venography findings. Tissue gel (NBCA; Compont Medical Devices Co, Beijing, China), coil (Cook Medical/Boston Scientific), or a combination of these two agents were the choices for VE if there was no spontaneous splenorenal shunt. Otherwise, coil alone or coil combined with tissue gel was recommended with the aim of reducing the risk of ectopic embolism. With respect to different conditions of esophageal and gastric varices (EGVs), the surgeon chose the embolic material according to the intraoperative contrast condition. Coils were applied when the maximum diameter of the EGV exceeds 8 ​mm or when a splenorenal shunt exists. For EGVs with a single varicose vein, single coil embolization was used as appropriate. When NBCA was used, NBCA and iodized oil were pressed at a 1:1.5 to 1:3.5 proportional dilution. The lower the concentration of NBCA, the slower the coagulation rate of blood. The specific proportion was determined by the diameter of the varicose vein, the area of the varicose vein plexus, and the flow rate. The microcatheter was rinsed with 5% glucose solution before embolization, to effectively prevent the NBCA from contacting with blood in the catheter. When the colloidal stasis was close to the tip of the catheter, the gel injection was stopped. The endpoint of VE was the disappearance of varices at post-embolization portal venography.

A 6- or 8-mm balloon was then used to dilate the liver parenchyma. Thereafter, a Viatorr stent (W.L. Gore and Associates) with a diameter of 8 ​mm was implanted. All patients started anticoagulation 24 ​h after TIPS implantation.

According to the embolic agent used in VE during TIPS, we divided the included patients into three groups: tissue gel (group A), combination (group B), and coil (group C) groups.

### Endpoints and follow-up

The primary endpoint was the 1-year rebleeding rate after TIPS creation. Recurrent VB was defined as gastroesophageal VB identified by endoscopy.^19^ The secondary endpoints included shunt dysfunction (defined as stent stenosis >50% or occlusion confirmed with Doppler ultrasonography or shunt venography, if necessary), overt hepatic encephalopathy (HE) that was diagnosed and graded according to the West Haven criteria, liver function assessment, total fluoroscopy and procedure time, and embolic agent-related expense.^20^^,^^21^ All patients were followed up in the clinic with clinical, biochemical, and color Doppler ultrasonography evaluations at 1, 3, and 6 months, and every 6 months thereafter. Patients were admitted to our department at any time once recurrent bleeding, HE, or other severe complications occurred. The follow-up period was defined as the time interval between TIPS creation and liver transplantation, death, or the last follow-up (August 2018).

### Statistical analysis

Continuous variables are summarized as the median with 95% confidence interval or the mean with standard deviation. Categorical variables are expressed as frequencies and percentages. Comparisons of variables among the three groups were performed using the analysis of variance, chi-square test, or Fisher test, as appropriate. Comparison of embolic agent-related expense was performed using the Kruskal-Wallis test. Comparison of total fluoroscopy time and procedure time was performed using the Scheffe test. Variables with P values ​≤ ​0.05 were considered statistically significant. All statistical analyses were performed using SPSS 18.0 for Windows (IBM Corporation, Somers, NY, USA).

## Results

### Patient characteristics

**Fig. 2:**
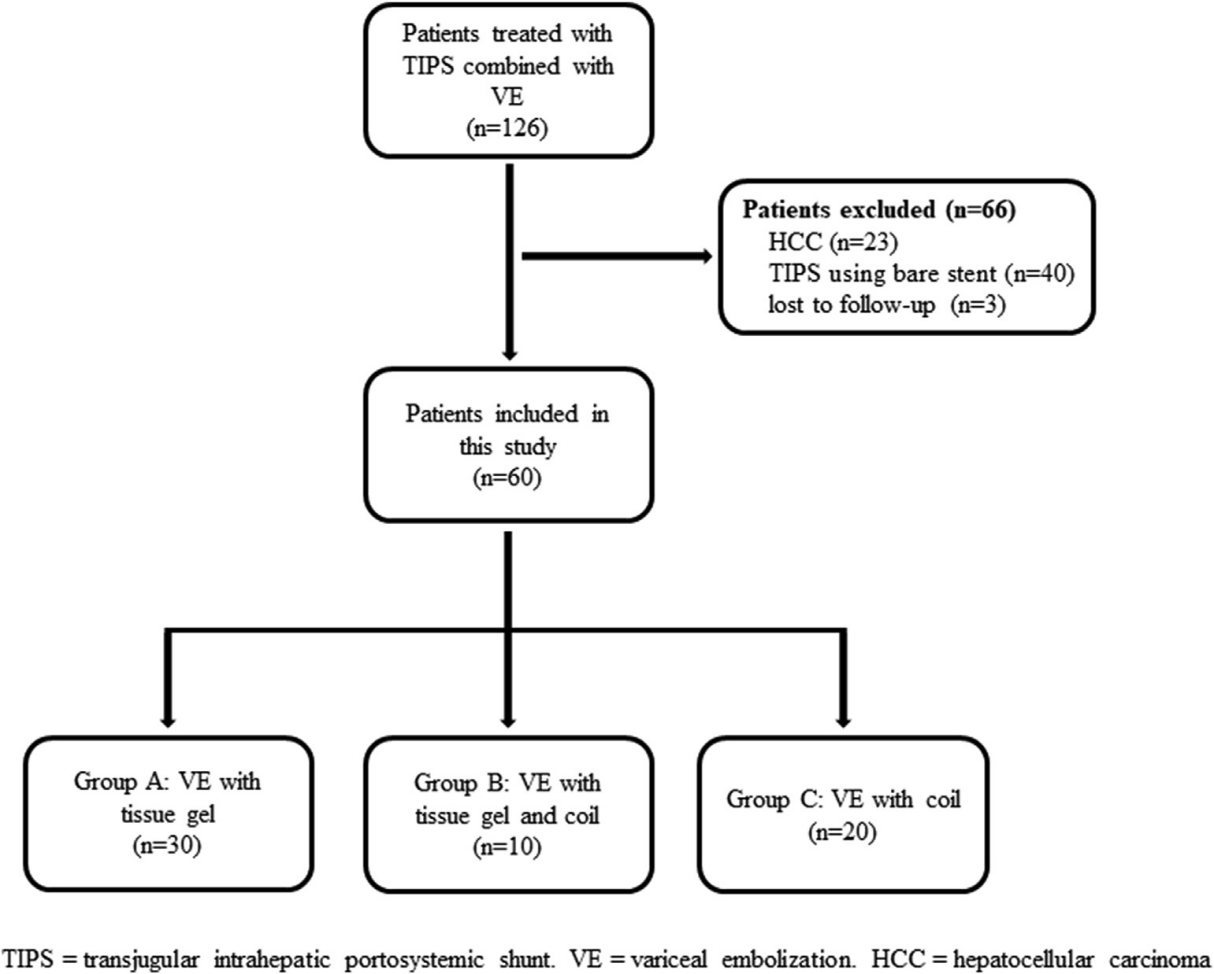
Study flowchart.

A total of 60 patients met the inclusion criteria and were finally included. The disposition of the patients is shown in Fig. 2. Of the patients, 44 and 16 were graded as GOV 2 and IGV 1, respectively. Tissue gel, combination of tissue gel and coil, and coil were used for VE during TIPS in 30, 10, and 20 patients, respectively. Twenty-nine (48.3%) patients had hepatitis B virus-related cirrhosis. Thirteen (21.7%) patients presented with portal vein thrombosis. The detailed baseline characteristics are shown in Table 1.

**Table 1:** Patient characterstics

Characteristics	Total (n ​= ​60)	Tissue gel group (n ​= ​30)	Combination group (n ​= ​10)	Coil group (n ​= ​20)	*P*
Age	59.7 ​± ​13.1	58 3 ​± ​14.0	59.8 ​± ​13.1	61.7 ​± ​12.1	0.677^a^
Sex					0.400^+^
Male	37 (61.7%)	18 (60.0%)	8 (80.0%)	11 (55.0%)	
Female	23 (38.3%)	12 (40.0%)	2 (20.0%)	9 (45.0%)	
Etiology of cirrhosis					0.640^#^
Hepatitis B	29 (48.3%)	15 (50.0%)	5 (50.0%)	9 (45.0%)	
Autoimmune Hepatitis	4 (6.7%)	2 (6.7%)	1 (10.0%)	1 (5.0%)	
Schistosome	9 (15.0%)	3 (10.0%)	3 (30.0%)	3 (15.0%)	
Others	18 (30.0%)	10 (33.3%)	1 (10.0%)	7 (35.0%)	
Variceal type					0.958^+^
GOV2	44 (73.3%)	22 (73.3%)	7 (70.0%)	15 (75.0%)	
IGV1	16 (26.7%)	8 (26.7%)	3 (30.0%)	5 (25.0%)	
CTP grade(n)					0.841^#^
A	35 (58.3%)	17 (56.7%)	5 (50.0%)	13 (65.0%)	
B	24 (40.0%)	12 (40.0%)	5 (50.0%)	7 (35.0%)	
C	1 (1.7%)	1 (3.3%)	0	0	
CTP score	6.4 ​± ​1.2	6.6 ​± ​1.4	6.6 ​± ​1.3	6.2 ​± ​1.0	0.455^a^
MELD score	11.2 ​± ​2.5	11.7 ​± ​2.4	11.9 ​± ​2.8	10.2 ​± ​2.0	0.071^a^
PPG pre-TIPS (mmHg)	32.5 ​± ​6.8	34.1 ​± ​7.2	29.8 ​± ​6.9	31.6 ​± ​5.8	0.178^a^
PPG post-TIPS (mmHg)	22.0 ​± ​5.8	23.6 ​± ​6.5	21.4 ​± ​4.9	19.8 ​± ​4.3	0.074^a^
Early TIPS	34 (56.7%)	21 (70.0%)	6 (60.0%)	7 (35.0%)	0.049^+^
Treatment history					0.909^#^
Band ligation	3 (5.0%)	2 (6.7%)	1 (10.0%)	0	
Injection sclerotherapy	2 (3.3%)	2 (6.7%)	0	0	
Splenectomy/PSE	6 (10.0%)	2 (6.7%)	1 (10.0%)	3 (15.0%)	
Portal vein punctured					0.536^+^
Left	39 (65.0%)	20 (66.7%)	5 (50.0%)	14 (70.0%)	
Right	21 (35.0%)	10 (33.3%)	5 (50.0%)	6 (30.0%)	
Stent length					0.074^#^
5cm	8 (13.3%)	1 (3.3%)	2 (20.0%)	5 (25.0%)	
6cm	24 (40.0%)	10 (33.3%)	5 (50.0%)	9 (45.0%)	
7cm	23 (38.3%)	14 (46.7%)	3 (30.0%)	6 (30.0%)	
8cm	5 (8.3%)	5 (16.7%)	0	0	
Follow-up period (months)	12.9 ​± ​7.7	10.3 ​± ​3.7	11.8 ​± ​7.9	17.7 ​± ​9.9	0.007^-^
PVTT	13 (21.7%)	7 (23.3%)	2 (20.0%)	4 (20.0%)	0.999^#^

a ANOVA; + Chi-square test; # Fisher test; - Kruskal-Wallis test.

Most of the patients underwent stent insertion as “early TIPS,” with 21 (70.0%), 6 (60.0%), and 7 (35.0%) patients in groups A, B, and C, respectively. The left and right branches of the portal vein were punctured in 39 (65.0%) and 21 (35.0%) patients, respectively. The length of the inserted Viatorr stents with diameter of 8 ​mm ranged from 5 to 8 ​cm (Table 1).

### Variceal rebleeding

During the median follow-up of 12.9 months (range 1–29 months), the 1-year variceal rebleeding rate was 11.7% (Table 2). Three (10%), zero (0%), and four (20%) patients experienced recurrent VB in groups A, B, and C, respectively. No statistical significance was observed in terms of the 1-year variceal rebleeding rate (P ​= ​0.254). Six of these seven patients died after recurrent VB, and no other death was observed within 1 year after TIPS creation. The 1-year mortality rate was 10%, 0%, and 15%, respectively. Similarly, no statistical significance was observed (P ​= ​0.533).

**Table 2:** Efficacy and safety comparison between tissue gel, coil, and combination groups.

Characteristics	Total (n ​= ​60)	Tissue gel group (A) (n ​= ​30)	Combination group (B) (n ​= ​10)	Coil group (C) (n ​= ​20)	*P*
Rebleeding 12 ​M after TIPS	7 (11.7%)	3 (10.0%)	0	4 (20.0%)	0.254^a^
Rebleeding 3 ​M after TIPS	3 (5.0%)	3 (10.0%)	0	0	0.277^a^
Rebleeding 3~6 ​M after TIPS	2 (3.3%)	0	0	2 (10.0%)	0.133^a^
Rebleeding 6–12 ​M after TIPS	2 (3.3%)	0	0	2 (10.0%)	0.133^a^
Died 12 ​M after TIPS	6 (10.0%)	3 (10.0%)	0	3 (15.0%)	0.533^a^
Died 3 ​M after TIPS	3 (5.0%)	3 (10.0%)	0	0	0.277^a^
Died 3~6 ​M after TIPS	2 (3.3%)	0	0	2 (10.0%)	0.133^a^
Died 6–12 ​M after TIPS	1 (1.7%)	0	0	1 (5.0%)	0.500^a^
Charge of Embolic Agents ($)	637.6	310.5	950.8	974.6	<0.001^-^
Complications	14 (23.3%)	10 (33.3%)	1 (10.0%)	3 (15.0%)	0.244^a^
Overt HE	9 (15.0%)	6 (20.0%)	0	3 (15.0%)	0.440^a^
Shunt dysfunction	2 (3.3%)	1 (3.3%)	0	1 (5.0%)	1.00^a^

TIPS ​= ​transjugular intrahepatic portosystemic shunt. HE ​= ​hepatic encephalopathy.

a Fisher test; - Kruskal-Wallis test.

### Shunt dysfunction

Stent dysfunction occurred in two patients (Table 2). One patient in group A developed acute occlusion caused by thrombus. The other patient in group C underwent stent stenosis during the follow-up. The latter patient experienced variceal rebleeding. Shunt patency was restored with TIPS revision in both of these two patients.

### Overt HE

During the follow-up, nine (15.0%) patients experienced overt HE (Table 2). Seven (77.8%) cases were categorized as West Haven grade II, and two cases (22.2%) were categorized as West Haven grade III. Six (20.0%), zero (0%), and three (15.0%) patients developed overt HE in groups A, B, and C, respectively (P ​= ​0.440). All patients recovered after medical treatment.

### Liver function assessment

Liver function was assessed according to the CTP score before the procedure and at every follow-up point. The mean CTP score gradually decreased during the follow-up. No liver failure occurred during the follow-up. The detailed liver function assessment is shown in Table 3.

**Table 3:** Child-Turcotte-Pugh score before and after TIPS creation in tissue gel, coil, and combination groups.

Groups	Pre-TIPS	1 ​M	3 ​M	6 ​M	12 ​M
Tissue gel group (A)	6.57 ​± ​1.35	6.30 ​± ​1.29	6.14 ​± ​0.85	6.11 ​± ​0.83	5.94 ​± ​0.82
Combination group (B)	6.60 ​± ​1.26	6.22 ​± ​1.30	5.89 ​± ​0.78	6.11 ​± ​1.05	5.91 ​± ​0.79
Coil group (C)	6.1 ​± ​0.99	6.1 ​± ​0.97	6.05 ​± ​0.78	6.06 ​± ​0.83	5.73 ​± ​0.59

TIPS ​= ​transjugular intrahepatic portosystemic shunt.

### Fluoroscopy time and procedure time

The mean total fluoroscopy time (33 ​± ​5 ​min for tissue gel, 41 ​± ​5 ​min for the combination material, and 42 ​± ​5 ​min for coil) was shorter in procedures using tissue gel. After the Scheffe test, statistical significance was observed between groups A and B (P ​< ​0.001) and between groups A and C (P ​< ​0.001) in terms of total fluoroscopy time. No statistical significance was observed between groups B and C (P ​= ​0.839).

The mean total procedure time (140 ​± ​34 ​min for tissue gel, 159 ​± ​32 ​min for the combination material, and 166 ​± ​32 ​min for coil) was also shorter in procedures using tissue gel. After the Scheffe test, statistical significance in procedure time was observed between groups A and C (P ​= ​0.036). No statistical significance was observed between groups A and B (P ​= ​0.307) and between groups B and C (P ​= ​0.876).

### Embolic agent-related expense

**Fig. 3:**
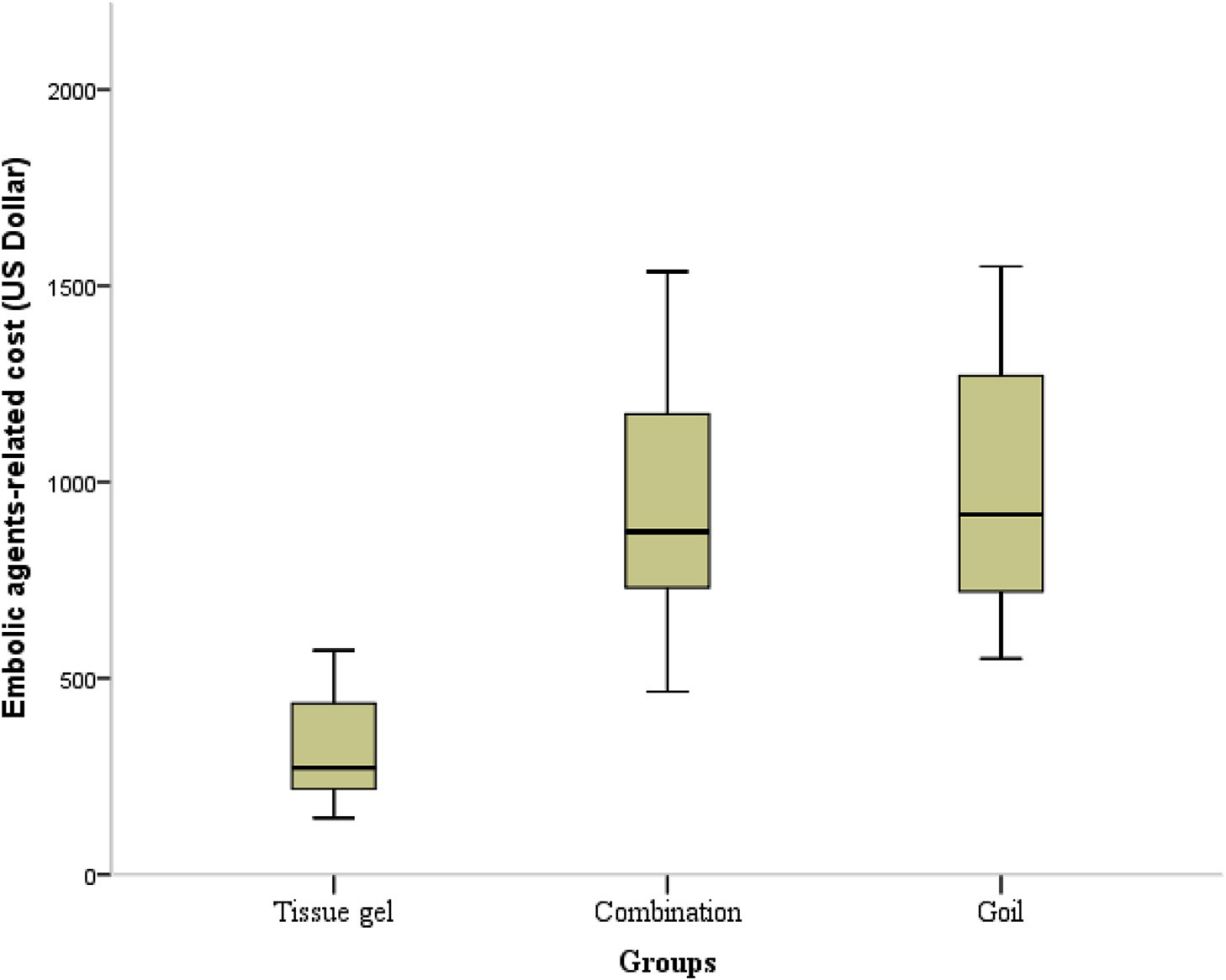
Embolic agent-related expense in the tissue gel, combination, and coil groups. After the Kruskal-Wallis test, the tissue gel group showed a significant lower cost than the coil group (P ​< ​0.001) and the tissue gel combined with coil group (P ​< ​0.001).

The mean embolic agent-related expense for groups A, B, and C were $310.5, $950.8, and $974.6, respectively (Fig. 3). After the K–W test, statistical significance in expense was observed between groups A and B (P ​< ​0.001) and between groups A and C (P ​< ​0.001). No statistical significance was observed between groups B and C (P ​= ​0.877). Tissue gel for VE during TIPS showed a significantly lower cost than coil and tissue gel combined with coil. No ectopic embolism occurred among all 60 patients.

## Discussion

With its identified treatment efficacy and safety for patients with VB and cirrhosis, TIPS has been well documented and endorsed by related guidelines and recommendations.^1^^,^^4^^,^^19^ The ideal treatment function of TIPS for VB is providing adequate decompression and minimizing the risk factors associated with variceal rebleeding, including remaining varices owing to their fragile nature.^9^ In addition, varices, especially cardiofundal varices (GOV 2 and IGV 1), are located distant from the TIPS and may be influenced by the preferential blood flow through a spontaneous portosystemic shunt after TIPS creation.^22^^,^^23^ Several previous studies have compared the treatment efficacy and safety of TIPS combined with VE versus TIPS alone for the management of VB, and most of them demonstrated that TIPS combined with VE had better treatment efficacy with respect to variceal rebleeding or stent patency.^9^, ^10^, ^11^, ^12^, ^13^, ^14^ Chen et al. performed a prospective randomized controlled trial and showed that the 6-month overall rate of shunt patency in the group with TIPS combined with VE was significantly higher than that in the TIPS-only group (96.2% vs. 82.0%, P ​= ​0.019)^10^. A similar result was also identified with respect to the 6-month overall rate of recurrent VB (5.7% vs. 20.0%, P ​= ​0.029).

Several embolic agents, such as tissue gel, coil, gelatin sponge, and vascular plug, have been used for VE during TIPS.^10^^,^^13^^,^^15^, ^16^, ^17^, ^18^ To our best knowledge, only one retrospective study that compared two kinds of embolic agents has been reported thus far.^18^ Sarwar et al. compared the treatment outcomes and material costs between vascular plugs and pushable coils for VE during TIPS.^18^ They found that the use of vascular plugs or coils for VB after TIPS has similar treatment outcomes in terms of variceal rebleeding and mortality. Nevertheless, coils have a significantly lower material cost than vascular plugs. The study identified that coil is a better choice for VE than vascular plug. As a solid material, coil has a favorable compliance and a controllable release process. Coil has been used for VE through different techniques.^9^^,^^15^

Tissue gel is also widely used for VE during TIPS, with NBCA as the preferred choice. NBCA is a watery solution that polymerizes and hardens within 20 ​s in a physiological milieu and instantaneously upon contact with blood. This characteristic makes NBCA ideal for obliterating vessels and controlling bleeding, and it has been successfully applied for the treatment of VB during the past 40 years.^24^ In addition, the use of NBCA has been extended to the treatment of ectopic varices in the jejunum, stoma, rectum, and duodenum.^25^

This study compared the treatment efficacy, safety, and embolic agent-related expense between tissue gel and coil for VE during TIPS. The results showed that tissue gel, coil, or tissue gel combined with coil had similar treatment outcomes with respect to variceal rebleeding, stent patency, overt HE, and liver function. Tissue gel has a significantly lower material cost than coil or tissue gel combined with coil ($310.5 vs. $974.6, P ​< ​0.01; $310.5 vs. $950.8, P ​< ​0.01). Although coil offers greater operator control owing to its detachable nature of deployment, all cases included in this study achieved complete occlusion with no serious adverse events. Considering treatment efficacy and value-based health care, tissue gel should be considered a better choice than coil for VE during TIPS.

Some points and tips should be mentioned about procedural skills in tissue gel usage. It is necessary to dilute the gel with Lipiodol in order to prevent the gel from quickly solidifying. Lipiodol is not only used for dilution but also for fluoroscopic monitoring of delivery during embolization. However, overdilution of the glue prolongs the polymerization process, which could increase the risk of ectopic embolism.^26^ On the basis of our experience, the optimal proportions for the gel-Lipiodol mixture are 1:1.5 to 1:3.5. A mixture with a lower proportion of gel is often used for varices with a smaller diameter, and vice versa. The ideal endpoint of embolization should be when the pushed gel extends to the catheter tip. It is preferable to perform VE with gel before balloon angioplasty and stent insertion, in order to achieve adequate and extensive obliteration of the varices and their feeding veins. In addition, pre-TIPS VE has the technical benefit of increased visualization of varices owing to variceal filling and decreased risk of nontarget systemic coil embolization, as no large-caliber shunt is present to allow systemic coil migration.^27^ The tissue gel must be slowly and continuously injected to achieve complete deposition in varices. If significant flow reflux to the portal vein is observed, injection must be immediately stopped and balloon or stent intervention should be performed to avoid pulmonary embolization and portal vein embolization.

Notably, all included patients in this study received the Viatorr stent, an ePTFE-covered device specially designed for TIPS.^6^^,^^7^^,^^28^ The Viatorr stent is reported to be superior to other covered stents, such as the Fluency, for TIPS creation and was approved to be available in China since 2016.^29^

This study had several limitations. First, the retrospective design could lead to selection bias. The selection of embolic agents in this study was mainly based on physician preference after variceal assessment. Therefore, the number of patients in the three cohorts was disproportionate. Further prospective randomized controlled trials are warranted on this topic, with reduced bias. Second, the sample size was small and the intermediate follow-up period limits long-term outcome assessment. Nevertheless, this study presents a positive attempt in comparing tissue gel and coil, which is currently lacking in the literature of this field. Finally, other embolic agents such as vascular plug and gelatin sponge were not included in the comparison in this study. Nevertheless, a prior study has identified that vascular plug achieves similar treatment outcome to that of coil, with a significantly higher material cost.^18^ Gelatin sponge is not as widely applied for VE during TIPS as tissue gel and coil.

In conclusion, tissue gel and coil have similar treatment efficacy and safety for VE during TIPS. Considering value-based health care, tissue gel should be preferred as an ideal embolic agent because of its significantly lower material cost. Further prospective trials with a large sample size and long-term follow-up period are warranted to validate our findings.

## Financial support

This study was funded by the Jiangsu Provincial Medical Talent Funding (ZDRCA2016038), the Suzhou Special Diagnosis and Treatment Technology of Clinical Key Diseases (LCZX201704), the 10.13039/501100001809National Natural Science Foundation of China (81771945, 81901847), the 10.13039/501100004608Natural Science Foundation of Jiangsu Province (BK20190177), and the Suzhou Science and Technology Youth Plan (KJXW2018003). Funding sources had no involvement in the financial support for the conduct of the research and preparation of the article.

GOV ​= ​gastro-esophageal varices. IGV ​= ​isolated gastric varices. CTP = Child-Turcotte-Pugh. MELD ​= ​model for end-stage liver disease. PPG ​= ​portosystemic pressure gradient. TIPS ​= ​transjugular intrahepatic portosystemic shunt. PSE ​= ​partial splenic embolization. PVTT ​= ​portal vein tumor thrombosis.

## Declaration of competing interest

The authors declared that they have no conflicts of interest to this work. We declare that we do not have any commercial or associative interest that represents a conflict of interest in connection with the work submitted.

## References

[1] Garcia-Tsao G., Abraldes J.G., Berzigotti A. (2017). Portal hypertensive bleeding in cirrhosis: risk stratification, diagnosis, and management: 2016 practice guidance by the American Association for the study of liver diseases. Hepatology.

[2] Garcia-Tsao G., Bosch J. (2010). Management of varices and variceal hemorrhage in cirrhosis. N Engl J Med.

[3] Tripathi D., Stanley A.J., Hayes P.C. (2015). U.K. guidelines on the management of variceal haemorrhage in cirrhotic patients. Gut.

[4] European Association for the Study of the Liver (2018). Electronic address eee, European Association for the Study of the L. EASL Clinical Practice Guidelines for the management of patients with decompensated cirrhosis. J Hepatol.

[5] Garcia-Pagan J.C., Caca K., Bureau C. (2010). Early use of TIPS in patients with cirrhosis and variceal bleeding. N Engl J Med.

[6] Vignali C., Bargellini I., Grosso M. (2005). TIPS with expanded polytetrafluoroethylene-covered stent: results of an Italian multicenter study. AJR American journal of roentgenology.

[7] Rossi P., Salvatori F.M., Fanelli F. (2004). Polytetrafluoroethylene-covered nitinol stent-graft for transjugular intrahepatic portosystemic shunt creation: 3-year experience. Radiology.

[8] Hausegger K.A., Karnel F., Georgieva B. (2004). Transjugular intrahepatic portosystemic shunt creation with the Viatorr expanded polytetrafluoroethylene-covered stent-graft. J Vasc Intervent Radiol : J Vasc Intervent Radiol.

[9] Tesdal I.K., Filser T., Weiss C. (2005). Transjugular intrahepatic portosystemic shunts: adjunctive embolotherapy of gastroesophageal collateral vessels in the prevention of variceal rebleeding. Radiology.

[10] Chen S., Li X., Wei B. (2013). Recurrent variceal bleeding and shunt patency: prospective randomized controlled trial of transjugular intrahepatic portosystemic shunt alone or combined with coronary vein embolization. Radiology.

[11] Gaba R.C., Bui J.T., Cotler S.J. (2010). Rebleeding rates following TIPS for variceal hemorrhage in the Viatorr era: TIPS alone versus TIPS with variceal embolization. Hepatology international.

[12] Yu J., Wang X., Jiang M. (2019). Comparison of transjugular intrahepatic portosystemic shunt (TIPS) alone and combined with embolisation for the management of cardiofundal varices: a retrospective study. Eur Radiol.

[13] Shi Y., Tian X., Hu J. (2014). Efficacy of transjugular intrahepatic portosystemic shunt with adjunctive embolotherapy with cyanoacrylate for esophageal variceal bleeding. Dig Dis Sci.

[14] Xiao T., Chen L., Chen W. (2011). Comparison of transjugular intrahepatic portosystemic shunt (TIPS) alone versus TIPS combined with embolotherapy in advanced cirrhosis: a retrospective study. J Clin Gastroenterol.

[15] Lee E.W., Saab S., Gomes A.S. (2014). Coil-Assisted retrograde transvenous obliteration (CARTO) for the treatment of portal hypertensive variceal bleeding: preliminary results. Clin Transl Gastroenterol.

[16] Romero-Castro R., Ellrichmann M., Ortiz-Moyano C. (2013). EUS-guided coil versus cyanoacrylate therapy for the treatment of gastric varices: a multicenter study (with videos). Gastrointest Endosc.

[17] Gwon D.I., Ko G.Y., Yoon H.K. (2013). Gastric varices and hepatic encephalopathy: treatment with vascular plug and gelatin sponge-assisted retrograde transvenous obliteration--a primary report. Radiology.

[18] Sarwar A., Esparaz A.M., Tapper E.B. (2017). Comparison of vascular plugs and pushable coils for variceal embolization after TIPS. AJR American journal of roentgenology.

[19] de Franchis R., Baveno V.F. (2010). Revising consensus in portal hypertension: report of the Baveno V consensus workshop on methodology of diagnosis and therapy in portal hypertension. J Hepatol.

[20] Cai J., Guo W., He C. (2015). Shunt dysfunction: is it suitable as the primary end point in transjugular intrahepatic portosystemic shunt trials?. J Hepatol.

[21] ter Borg P.C., Hollemans M., Van Buuren H.R. (2004). Transjugular intrahepatic portosystemic shunts: long-term patency and clinical results in a patient cohort observed for 3-9 years. Radiology.

[22] Saad W.E. (2014). Combining transjugular intrahepatic portosystemic shunt with balloon-occluded retrograde transvenous obliteration or augmenting TIPS with variceal embolization for the management of gastric varices: an evolving middle ground?. Semin Intervent Radiol.

[23] Sanyal A.J., Freedman A.M., Luketic V.A. (1997). The natural history of portal hypertension after transjugular intrahepatic portosystemic shunts. Gastroenterology.

[24] Lunderquist A., Borjesson B., Owman T. (1978). Isobutyl 2-cyanoacrylate (bucrylate) in obliteration of gastric coronary vein and esophageal varices. AJR American journal of roentgenology.

[25] Choi J.W., Kim H.C., Jae H.J. (2015). Transcatheter embolotherapy with N-butyl cyanoacrylate for ectopic varices. Cardiovasc Intervent Radiol.

[26] Seewald S., Sriram P.V., Naga M. (2002). Cyanoacrylate glue in gastric variceal bleeding. Endoscopy.

[27] Lipnik A.J., Pandhi M.B., Khabbaz R.C. (2018). Endovascular treatment for variceal hemorrhage: TIPS, BRTO, and combined approaches. Semin Intervent Radiol.

[28] Maleux G., Nevens F., Wilmer A. (2004). Early and long-term clinical and radiological follow-up results of expanded-polytetrafluoroethylene-covered stent-grafts for transjugular intrahepatic portosystemic shunt procedures. Eur Radiol.

[29] Saad W.E., Darwish W.M., Davies M.G. (2010). Stent-grafts for transjugular intrahepatic portosystemic shunt creation: specialized TIPS stent-graft versus generic stent-graft/bare stent combination. J Vasc Interv Radiol.

